# Identification of breast cancer susceptibility genes using whole exome sequencing

**DOI:** 10.1186/1897-4287-10-S2-A41

**Published:** 2012-04-12

**Authors:** E Thompson, M Doyle, J Ellul, J Li, I Campbell

**Affiliations:** 1 Peter MacCallum Cancer Centre, East Melbourne, Australia

## 

Recent advances in technology have opened up the possibility of using next generation sequencing to efficiently uncover predisposing mutations in individuals with inherited cancer in an unbiased manner. We are conducting whole exome sequence analysis of germline DNA from multiple affected relatives from breast cancer families with the aim of identifying rare protein truncating and non-synonymous variants that are likely to include novel cancer predisposing mutations. Data from >70 exomes show that on average each individual only carries 30-50 protein truncating mutations and 300-400 rare non-synonymous missense variants. By considering only those variants shared by multiple affected relatives the number of candidate predisposing mutations can be dramatically reduced to just 3-5 truncating mutations and 10-20 nonsynonymous variants per family. Among the first 10 breast cancer families studied in detail, two harbour mutations in known breast cancer genes that were missed by clinical genetic testing either because the index case was a phenocopy who did not carry the mutation (*BRCA2*) or because the gene is not routinely tested in the context of breast cancer without additional clinical manifestations (*PTEN*). Among the remaining families candidate genes are currently being assessed for segregation among family members and for prevalence among an addition 800+ unexplained breast cancer families. In particular, we found truncating mutations in two genes that are involved in well-recognised DNA repair pathways but have not previously been associated with an increased risk of breast cancer. In summary, whole exome sequencing of multiple individuals from within each cancer family is proving to be an efficient strategy for rapidly identifying novel familial predisposing mutations.

